# Efficacy of a hybrid online training for panic symptoms and agoraphobia: study protocol for a randomized controlled trial

**DOI:** 10.1186/1745-6215-15-427

**Published:** 2014-11-04

**Authors:** Lara Ebenfeld, Stefan Kleine Stegemann, Dirk Lehr, David Daniel Ebert, Hooria Jazaieri, Wouter van Ballegooijen, Burkhardt Funk, Heleen Riper, Matthias Berking

**Affiliations:** Division of Health Training Online, Leuphana University Lueneburg, Innovation Incubator, Rotenbleicher Weg 67, 21335 Lueneburg, Germany; Chair of Clinical Psychology and Psychotherapy, Friedrich-Alexander-University Erlangen-Nuremberg, Bismarckstraße 1, 91054 Erlangen, Germany; Department of Psychology, Institute of Personality and Social Research, University of California, Berkeley, 4152 Tolman Hall, Berkeley, California 94720-1650 USA; Institute for Health and Care Research (EMGO), VU University Medical Center, Van der Boechorststraat 7, 1081, BT Amsterdam, The Netherlands; Faculty of Psychology and Education, VU University Amsterdam, Van der Boechorststraat 1, 1081, BT Amsterdam, The Netherlands

**Keywords:** Panic disorder, Agoraphobia, Sub-clinical, Internet, Mobile, Smartphone, Hybrid

## Abstract

**Background:**

Recently, internet-based interventions have been proposed as effective treatments for people with panic disorder (PD). However, little is known about the clinical effects of integrating mobile technology into these interventions. Because users carry their smartphones with them throughout the day, we hypothesize that this technology can be used to significantly support individuals with monitoring and overcoming their PD symptoms. The aim of the present study is to evaluate the efficacy and cost-effectiveness of a newly developed hybrid intervention that combines internet/PC with smartphone delivery to treat the symptoms of PD. The intervention is based on cognitive behavioral therapy and consists of six modules over a total of six weeks.

**Methods/Design:**

A two-arm randomized controlled trial (RCT) will be conducted to evaluate the effects of a hybrid online training module for PD. Based on a power calculation (d =0.60; 1-β of 80%; α =0.05), 90 participants with mild to moderate panic symptoms with or without agoraphobia (as assessed by the Panic and Agoraphobia Scale) will be recruited from the general population and randomly assigned to either the intervention group or a six-month waitlist control group. The primary outcome measure will be the severity of panic symptoms. Secondary outcomes will include depression, quality of life, and an observer-based rating of panic severity. Furthermore, data regarding acceptance and the usability of the smartphone app will be assessed. Assessments will take place at baseline as well as eight weeks, three months, and six months after randomization. Moreover, a cost-effectiveness analysis will be performed from a societal perspective. Data will be analyzed on an intention-to-treat basis and per protocol.

**Discussion:**

To our knowledge, this RCT is one of the first to examine the efficacy of a hybrid online training for adult PD. This study seeks to contribute to the emerging field of hybrid online training. If the intervention is efficacious, then research on this hybrid online training should be extended. The cost-effectiveness analysis will also indicate whether online training is an economical tool for treating PD among adults.

**Trial registration:**

German Clinical Trial Register: DRKS00005223 (registered on 15 August 2013).

## Background

Panic disorder (PD) is characterized by recurrent, unexpected panic attacks and a persistent worry about future panic attacks [1]. With a 12-month prevalence of 1.8% among adults, PD is one of the most common anxiety disorders [2]. In addition, sub-threshold cases have been estimated to be even more prevalent, with between 10 and 16% of the population experiencing a panic attack at some point during their lifetimes [3]. PD (with or without agoraphobia) is associated with a high psychiatric comorbidity, lower quality of life, and severe work impairment [4, 5], and also places a significant burden on healthcare systems [2, 6].

Psychotherapeutic treatments such as cognitive behavioral therapy (CBT) are effective for PD [7]. However, only 16.7% of people who suffer from anxiety disorders seek help from a mental health professional, and of these individuals, only 21.3% receive CBT, which is arguably the most effective treatment [8]. The reasons for this low endorsement of effective PD treatments include a lack of psychoeducation, a fear of stigmatization, and structural barriers such as a lack of access to adequate treatments [9, 10].

Interventions delivered via the internet have been proposed as a helpful method to overcome such barriers and facilitate access to empirically validated treatments [11, 12]. Although internet-based interventions based on CBT are effective at treating adult PD [13–16], one drawback of this method (compared with traditional face-to-face treatments) is the lack of direct support from a therapist throughout the course of treatment, particularly during exposure exercises. This lack of direct support from a therapist might increase the risk of clients not fully complying with the treatment, or dropping out of treatment completely. Studies that evaluate online trainings with self-exposure elements consistently report particularly high dropout rates [17, 18].

One potentially promising method to enhance the adherence to, and efficacy of, internet-based interventions for PD might be to complement interventions with components that are delivered through clients’ smartphones. Mobile components might assist patients in overcoming several limitations of traditional desktop- and laptop-based interventions. For example, these barriers might include situations in which the clients start to engage in exposure exercises in their natural environment (as is typical for *in vivo* exposure exercises) and are subsequently required to leave their desktop PCs or laptop. This results in a dilemma - either exposure exercises are exclusively conducted at the client’s desk or they are conducted without the device and therefore lack support during the exposure exercises. Consequently, clients are more likely to disengage from the intervention. In contrast, clients often carry their smartphones with them in almost any situation, and they might support clients in successfully completing the intended exposure exercise [19–21]. Moreover, when desktop PCs or laptops are used to monitor symptoms of PD to identify factors that cue panic attacks and avoidance, clients typically use daily or weekly electronic diary entries, which are likely biased by memory effects because clients must complete these diaries retrospectively and not in real-time [22–28]. In contrast, a mobile-based PD intervention tool can be used to assess the symptoms of PD in real-time through ecological momentary assessment (EMA) approaches [29–33]. Finally, a PC-based program can only prompt appropriate coping responses when the PC is on and clients are near it. Therefore, it is not available in other situations of their daily lives in which they inevitably encounter stimuli that trigger the symptoms of PD. In contrast, a mobile device that is nearly always on, or near, the client can be used as an ecological mobile intervention (EMI) device and prompt coping responses, potentially those that have been specifically identified as effective for the individual based on the EMA function of the device. Despite these advantages, no data are currently available regarding the efficacy of online-based interventions for PD that integrate a mobile component.

We developed the GET.ON PANIC intervention for adult PD. This intervention integrates desktop and mobile components into a hybrid online intervention based on CBT for PD. The desktop component is primarily used to provide text- and video-based psychoeducation, as well as exercises that require participants to write extensive texts (for example, in a cognitive restructuring module), which is difficult to do on smartphones. The mobile component is used to guide clients through self-monitoring and self-exposure tasks. To evaluate the efficacy and cost-effectiveness of GET.ON PANIC, we will conduct a randomized controlled trial (RCT).

## Methods/Design

### Study design

**Figure 1:**
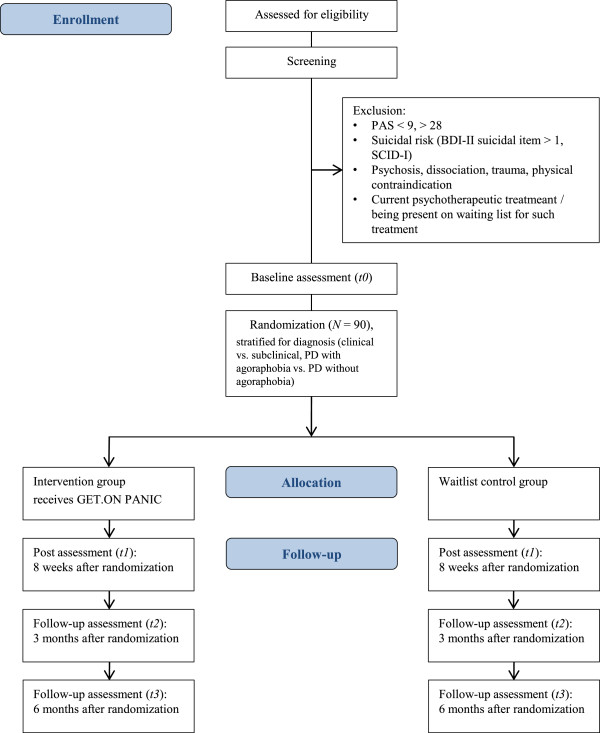
**Overview of study procedure.** BDI-II, Beck Depression Inventory II; PAS, Panic and agoraphobia scale; SCID-I, Structured clinical interview for DSM-IV Axis I Disorders.

We will conduct an RCT with two arms: an internet-based self-help intervention supported by a mobile application with minimal guidance from a coach (GET.ON PANIC), and a waitlist control group who will receive the intervention after a six-month follow-up assessment. Assessments will be conducted prior to randomization, at post-treatment (eight weeks), as well as at the three- and six-month follow-up assessments (see Figure 1). The Ethical Committee of Marburg approved this study (number: 2013-23 K), and it was registered with the German Clinical Trial Register (registration number: DRKS00005223).

### Study population

The study population will consist of a community sample of adults who suffer from mild to moderate panic symptoms. The inclusion criteria are the following: experiencing mild to moderate panic symptoms as assessed by the Panic and Agoraphobia Scale (PAS, score range: 9 to 28) [34, 35], being 18 years or older, having panic as the primary concern for seeking help, and having internet and smartphone access. Although both iOS™ and Android™ devices will be supported, the GET.ON PANIC APP will not run on entry-level smartphones with small screens and low memories. Therefore, the minimum system requirements are an iPhone™ 3GS or a comparable Android device. With respect to the operating system, iOS 6, iOS 7, and Android 2.3 or newer will be supported. Furthermore, because the mobile application periodically uploads data to our servers, we recommend that participants have a data plan to avoid unnecessary costs. The exclusion criteria for this study are the following: experiencing too mild (PAS score 0 to 8) or too severe (PAS score 29 to 52) panic symptoms; receiving current psychological help for anxiety problems or being on a waitlist for psychotherapy; having physical health problems assessed via self-report that prevents participants from engaging in self-exposure, as recommended by an established German guideline for treating people with PD and agoraphobia [36]; currently having posttraumatic stress disorder or psychotic or dissociative disorders assessed via self-report and clinical interview; and having current suicidality, as measured by a score above 1 on item 9 of the Beck Depression Inventory II (BDI-II) [37, 38] and question A9 of the Structured Clinical Interview for DSM-IV Axis I Disorders (SCID-I) [39]. In the event that potential participants are excluded because of suicidal ideation or intention, the exclusion procedure will be managed as determined by an established suicide protocol. All excluded participants will be contacted via email and provided with information regarding where they can obtain appropriate help.

### Sample size

The sample size for this study is based on the meta-analysis of self-help treatments for anxiety disorders by Haug *et al*. [13]. An effect size of d =0.83 was derived for PD after comparing the self-help group with the waitlist/placebo group. Because hybrid online training was not integrated into the meta-analysis, we chose a conservative estimation of d =0.60. To examine the efficacy of GET.ON PANIC with a two-tailed t-test (α =0.05; 1-β =0.80), a sample of 45 participants will be needed for each group. Thus, the total sample size for this study will be 90 participants.

### Randomization

We will use the computer program DatInf RandList Version 1.2 (DatInf GmbH, Tübingen, Germany) [40] to randomize participants into either the intervention group or the waitlist control condition. This random assignment will be stratified for clinical or subclinical symptomatology, as well as the presence or absence of agoraphobia.

### Procedure

Participants will be recruited from the general population via an online health center website postings in anxiety- and panic-related online forums, and newspaper articles about the research project. Participants will receive an email with detailed information about the study. Afterwards, participants will be invited to complete a screening questionnaire to evaluate their study eligibility. Participants will then have access to the online training platform via their email address (as their username) and a self-selected password. If participants meet the eligibility criteria, they will receive an ID number. Participants can then opt into study participation by reading, signing, and returning the informed consent document. Participants will then receive a link to complete the baseline questionnaires. After completing the baseline questionnaires (t0), participants will be invited to take part in a telephone interview. This interview has two purposes. The first is for a trained interviewer to conduct a diagnostic interview (SCID-I) to provide a detailed sample description [41, 42]. The second is to conduct an observer rating of anxiety symptoms using the Hamilton Anxiety Scale (HAM-A) [43] to strengthen the robustness of the self-report measures. Assessors blind to the participant treatment condition will conduct all observer-based ratings. The post-treatment measurement (t1) will be assessed eight weeks after randomization. Follow-up measures will be conducted at three months (t2) and six months (t3) after randomization. All questionnaires at the baseline, post- and follow-up assessments are self-reported and conducted via the internet, with the exception of the observer rating HAM-A, which will be performed at t0 and t1 via telephone. The waitlist control group will receive the treatment after t3.

### Intervention

**Figure 2:**
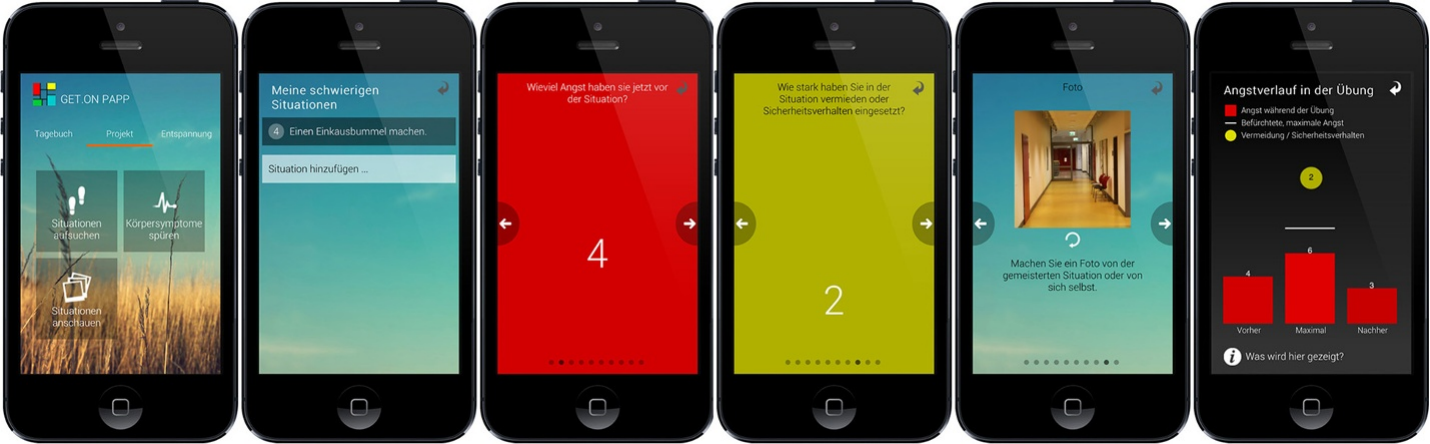
**App screenshots of GET.ON PANICAPP.** The app supports in vivo exposures. Participants start with ranking their anxiety provoking situations in a hierarchical order. Furthermore, they are asked to answer questions about their anxiety before and after performing the exposure exercise (for example the level of anxiety, the degree of avoidance and the severity of body symptoms). In addition, they have the ability to take a photo after an in vivo exposure exercise is completed. The bar chart in the end offers graphical feedback to the participants regarding the exposure performance.

GET.ON PANIC is a hybrid internet-based self-help intervention with minimal therapeutic guidance based on CBT principles [18, 44–46]. The hybrid online training consists of two components: a browser-based section (desktop PC or laptop) and a mobile application (smartphone app). The intervention is divided into six modules: psychoeducation, interoceptive exposure, *in vivo* exposure, two modules of cognitive restructuring, and relapse prevention (see Table 1). Using responsive web design, participants can use the program on a desktop PC, a laptop, a tablet, or a smartphone. An integrated read-aloud function allows participants to follow the lessons via audio narration. The app addresses interoceptive and *in vivo* exercises as well as diary and relaxation exercises. Detailed information about the development of the app can be found in the paper by Kleine Stegemann *et al*. [47].In the first module, participants will receive an overview of the different modules and the practical procedure of the online training. Information about PD will be provided, and personal goals will be defined. In addition, a mobile diary will be introduced to the participants. The emphasis of the second module is interoceptive exposure. The theoretical background of the relationship between bodily symptoms and anxiety will be provided in an interactive way with videos and writing exercises. The app contains three interoceptive exposure packages, each consisting of four different tasks. In module three, participants will rank their individual anxiety provoking situations in a hierarchy before beginning the app-assisted *in vivo* exposures (see Figure 2). In addition, participants will continue with the second of the interceptive exposure exercises, which address dizziness.

**Table 1:** **Overview of sessions**

Week	Content and homework	
**1**	Browser:	Psychoeducation:
		Information about panic
		Defining goals of training
		Setting up a reward list
	Mobile:	Daily diary
		Registration of current panic event (event-based)
		Daily summary of panic, avoidance, and mood
**2**	Browser:	Interoceptive exposure:
		Bodily symptoms in panic
		Avoidance
		Safety behaviors
	Mobile:	Respiratory interoceptive exposure exercises
		Daily diary
**3**	Browser:	*In vivo* exposure:
		Defining an anxiety hierarchy
	Mobile:	*In vivo* exposures
		Dizziness interoceptive exposure exercises
		Daily diary
**4**	Browser:	Cognitive restructuring I:
		Negative automatic thoughts
		Defining anxiety project (training schedule for exposures)
	Mobile:	*In vivo* exposures
		Further interoceptive exposure exercises
		Daily diary
**5**	Browser:	Cognitive restructuring II:
		Reality testing of automatic negative thoughts
	Mobile:	*In vivo* exposures
		Further interoceptive exposure exercises
		Daily diary
**6**	Browser:	Relapse prevention:
		Early warning signs
		Critical life events
		Evaluation of training and its aims
	Mobile:	Breathing and muscle relaxation exercises

The fourth module concerns cognitive restructuring. Participants will deal with the maladaptive relationships among situations, cognitions, and emotions. In addition, participants will continue with interoceptive and *in vivo* exposures. In module five, participants will engage in advanced cognitive restructuring, where they analyze their thinking patterns, identify their cognitive distortions and thinking errors, challenge their thoughts associated with panic, and replace their maladaptive thoughts with more constructive cognitions. Participants will continue to practice interoceptive and *in vivo* exposures for homework. The sixth and final module is related to relapse prevention. Participants will have the opportunity to reflect, summarize, and evaluate the online training as well as their progress toward achieving their predefined goals. Participants will work towards coping prior to experiencing anxiety-provoking events that might occur in the future by making two plans: one to identify early warning signs and one for dealing with difficult life circumstances. Furthermore, participants will be trained in breathing and muscle relaxation exercises to learn adequate coping strategies for daily stressors. Audio-based relaxation exercises will be available to the participants in both short and long formats on the app.

Throughout the study, participants will receive online technical support from an IT specialist to install the app, access to a personal online coach (a trained psychologist) to answer any questions, reminder messages for homework assignments, and brief homework feedback from the online coach based on a manual written by the first author (LE). All online coaches will receive online training with regard to GET.ON PANIC by the first and second authors (LE and SKSt), and will be supervised by licensed and experienced psychotherapists when offering guidance. The total amount of coaching time per participant will be approximately three hours for the duration of the six-week online training.

### Instruments

For an overview of the instruments at screening, baseline, after treatment, and at the follow-up assessments, see Table 2.

**Table 2:** **Overview of instruments per time of assessment**

Assessments	Screening	T0	T1	T2	T3
Sociodemographic data	**x**	**-**	**-**	**-**	**-**
Suicidality (Item 9, BDI-II)	**x**	**-**	**-**	**-**	**-**
Diagnosis (SCID-I, sections for anxiety disorders and current depressive episode)	**-**	**x**	**-**	**-**	
Panic and agoraphobia severity, self-rating (PAS)	**x**	**x**	**x**	**x**	**x**
Panic and agoraphobia severity, observer-rating (HAM-A)	**-**	**x**	**x**	**-**	**-**
Agoraphobic cognitions (ACQ)	**-**	**x**	**x**	**x**	**x**
Body sensations (BSQ)	**-**	**x**	**x**	**x**	**x**
Agoraphobic avoidance (MI)	**-**	**x**	**x**	**x**	**x**
Depressive symptoms (CES-D)	**-**	**x**	**x**	**x**	**x**
Quality of life (EQ-5D, SF-12)	**-**	**x**	**x**	**x**	**x**
Economic evaluations (TiC-P)	**-**	**x**	**-**	**-**	**x**
Negative effects of online health trainings	**-**	**-**	**(x)**	**(x)**	**(x)**
Attitudes towards seeking psychological help	**-**	**-**	**(x)**	**-**	**-**
User satisfactory	**-**	**-**	**(x)**	**-**	**-**
Technology acceptance of smartphone app	**-**	**-**	**(x)**	**-**	**-**
Usability of smartphone app (SUS)	**-**	**-**	**(x)**	**-**	**-**

T0 = Baseline, T1 = 8 weeks, T2 = 3 months, T4 = 6 months.

Assessments: x = intervention and control group, (x) = intervention group only.

ACQ, Agoraphobic cognitions questionnaire; BDI-II, Beck Depression Inventory II; BSQ, Body sensations questionnaire; CES-D, Center for epidemiological studies depression scale; EQ-5D, EuroQol; HAM-A, Hamilton anxiety scale; MI, Mobility inventory; PAS, Panic and agoraphobia scale; SCID-I, Structured clinical interview for DSM-IV Axis I Disorders; SF-12, Short form 12; SUS, System Usability Scale; TiC-P, Trimbos and the Institute of Medical Technology Assessment Cost Questionnaire for Psychiatry.

#### Screening and diagnostic interview

The preliminary screening will collect demographic data from participants, including their age, gender, education, therapeutic experience, and medication use, and the PAS will be administered (for a detailed description see the section regarding the primary outcome measure) [34, 35]. Item 9 of the BDI-II [37, 38] will be used to screen for suicidality. The BDI-II is a 21-item assessment of depressive symptoms that has demonstrated high internal consistency using outpatient samples [37].

The SCID-I [39] will be used to assess the presence of PD, agoraphobia, other anxiety disorders, and current depressive episodes. A trained interviewer will perform the interview via telephone. Previous studies have demonstrated the validity of telephone-based SCID-I interviews [41, 42].

### Primary outcome measure

#### Panic severity

The primary outcome will be the severity of panic and agoraphobia symptoms as assessed by the total PAS score [34, 35, 48]. This questionnaire was originally developed as a self- and observer-rating scale. In this study, we will use the self-rating questionnaire that was adapted into an online version. The PAS consists of 13 items grouped into five subscales, and an extra item regarding unexpected versus expected panic attacks. The five subscales assess the following areas: panic attacks, agoraphobic avoidance, anticipatory anxiety, daily life limitations, and health concerns (for example, the fear of physical harm or the fear of an organic cause). These subscales can be evaluated separately or as a total score that combines all subscales, ranging from 0 to 52 points. A higher score on the PAS indicates more panic symptoms. The psychometric properties of the scale are satisfactory, with a Cronbach’s alpha of 0.86 [49]. A score of between 0 and 8 indicates no clinically relevant symptoms, scores of between 9 and 28 indicate moderate symptoms, and a score of 29 or higher indicates a severe level of symptoms [49].

### Secondary outcome measures

#### Depression

Depressive symptoms will be measured using the Allgemeine Depressions-Skala (ADS) [50], the German adaptation of the Center for Epidemiological Studies Depression Scale (CES-D) [51]. The ADS consists of 20 items that refer to the previous week and are answered using a four-point Likert scale. The total score ranges from 0 to 60. Its internal consistency is α =0.89, and its split-half reliability is r =0.91 [50].

#### Quality of life

Quality of life will be measured using the EuroQol (EQ-5D) [52] and the Short Form 12 (SF-12) [53]. The EQ-5D is a well-established measurement of quality of life that consists of five items assessing mobility, self-care, common activities, pain and/or discomfort, and anxiety and/or depression, as well as a visual analogue scale concerning health status. We will also use the SF-12, which consists of 12 items that assess eight health domains: physical functioning, role limitations, pain, general health perception, vitality, mental health, emotional role, and social functioning. The SF-12 generates two summary scores: physical health and mental health.

#### Agoraphobic cognitions

Agoraphobic cognitions will be measured using the Agoraphobic Cognitions Questionnaire (ACQ) [54, 55]. The ACQ consists of 14 items, and its total score ranges from 14 to 70. Craske *et al*. reported that the ACQ has an internal reliability of α =0.80 [56].

#### Bodily sensations

The Body Sensation Questionnaire (BSQ) [54, 55] is a 17-item self-report questionnaire that measures bodily sensations. The BSQ ranges from 17 to 85 points and has a satisfactory internal reliability of α =0.87 [54].

#### Agoraphobic avoidance

The Mobility Inventory (MI) [54, 55] measures agoraphobic avoidance. The MI consists of 27 items that address the most important agoraphobic situations. Each item is rated both for when patients are alone and when they are accompanied. These two scales have a summed score ranging from 27 to 135 points, respectively. The internal consistencies are α =0.94 (alone) and α =0.91 (accompanied) [57].

#### Observer rating anxiety symptoms

In addition, the secondary outcome measures will include the observer-rated HAM-A [43, 58] to obtain a more detailed understanding of symptom severity. The HAM-A is a 14-item clinician-reported rating scale with a total score ranging from 0 to 30. Thus, we will use the structured interview guide for the HAM-A (SIGH-A) [59]. The interview has shown satisfactory inter-rater and test-retest reliabilities of Intraclass Correlation Coefficients of respectively 0.99 and 0.89 [59].

#### Economic evaluation

We will use an adaption of the Trimbos/iMTA questionnaire to measure the costs associated with psychiatric illness (TiC-P) [60] with regard to the German healthcare system.

#### Diary data

EMA data will be collected via the mobile application GET.ON PANIC APP [47]. This app contains a diary where clients can record their panic attacks and monitor their progress using daily summaries. With regard to the latter, clients will summarize their general anxiety levels, their degree of avoidance, and their moods each evening. The application also records the type and number of exposure exercises performed by the client.

#### Additional measures

We will also collect data concerning technology acceptance (via a questionnaire based on the technology acceptance model; TAM) [61, 62], the usability of the smartphone app GET.ON PANIC APP (via the System Usability Scale; SUS) [63, 64], user satisfaction of the online training (a self-designed questionnaire based on the German version of the Client Satisfaction Questionnaire [65, 66], and the adverse effects of psychotherapy [67].

### Statistical analyses

The analyses will be performed based on the Consolidated Standards of Reporting Trials (CONSORT) statement regarding eHealth [68]. The data will be analyzed on an intention-to-treat basis. We will also conduct per protocol and completers-only investigations as secondary analyses.

#### Treatment efficacy

Group differences in the baseline values of the primary outcome will be compared using t-tests to assess whether randomization was successful. Missing data will be addressed following the recommendations of Little and Rubin [69] and Schafer [70]. We will analyze the PAS data at eight weeks post-treatment using between-group analyses of covariance with regard to the individual baseline PAS scores, adjusted for sex, age, and socioeconomic status. We will use Cohen’s d to measure the between-group effect size. Cohen’s d will be calculated as the difference between the mean post-test scores of the intervention group and the control group divided by the pooled standard deviation [71]. All other secondary outcomes will be analyzed in a similar manner.

We will also conduct clinical significance change analyses as described by Jacobson and Truax [72]. In the first step, we will test whether the changes from pre-test to post-test are statistically reliable and build a reliable change index (RCI). In the second step, we will calculate clinical significance. Based on the RCI, the participants who display a reliable positive change, no change, or a reliable deteriorated change will be classified as responders, non-responders, or deteriorated, respectively [72]. We will use mixed-model regressions to examine the long-term effect on the primary outcome after three and six months.

#### Economic analyses

We will conduct an economic evaluation by performing a cost-effectiveness analysis (CEA) and a cost-utility analysis (CUA) from a societal perspective. The clinical outcome of the CEA will be the severity of panic symptoms as assessed by the PAS. Quality-adjusted life years (QALYs) will be calculated for the CUA. A non-parametric bootstrapping method with 95% confidence intervals will be used to assess the differences between the intervention and control groups. The intervention and waitlist control groups will be compared in terms of incremental costs and incremental effects. Thus, we will calculate the incremental cost-effectiveness ratio (ICER). Bootstrapping via 5,000 iterations will be used to quantify the uncertainty in the ratios and to test the robustness of the ICER. The results will be displayed on a cost-effectiveness plane and via a cost-effective acceptability curve. A multi-way sensitivity analysis will be conducted to test the robustness of the base-case findings. For instance, these analyses will integrate data based on the EMA to reduce the retrospective bias of self-reported panic symptom severity.

## Discussion

Given the availability and the daily use of smartphones, mobile-based health interventions have become increasingly popular [73–78]. Evidence for the efficacy of these studies comes from attempts to promote physical activity [79], cope with schizophrenia [25], and overcome child anxiety [80]. Despite the obvious advantages of integrating mobile components into online-based treatments (for example, mobile components can be used for EMA, supporting exposure exercises away from home, and EMI), no data are available regarding the efficacy of such an intervention for PD. Thus, we developed the hybrid online training GET.ON PANIC based on CBT, and we will test the efficacy and cost-effectiveness of this online training among people with clinical or subclinical PD with or without agoraphobia at post-assessment, as well as at three- and six-month follow-ups, compared with a waitlist control group who will receive the online training after the last assessment, but who will have free access to the usual treatments.

This study has several limitations. First, the absence of an active control group does not allow us to clarify the mechanisms responsible for changes in treatment. However, because this study is one of the first to test the efficacy of hybrid online training for PD, evaluating the efficacy of the intervention is an important research question in and of itself. If the present study provides evidence regarding the efficacy of GET.ON PANIC, then future research should detect the mechanisms that drive the change within the intervention. Such research should also include dismantling studies that compare online-based interventions with or without a mobile component to explain the additional benefits of including a mobile component. Second, using a waitlist control group design does not enable a comparison between newly developed hybrid online training and the current gold standard (face-to-face CBT to treat people with PD and agoraphobia). Future studies should compare hybrid online training with the gold standard. Finally, this study is not designed to test the long-term efficacy of GET.ON PANIC. With an assessment time of six months, we will only be able to make conclusions over a relatively short period of time. A 12-month follow-up assessment is desirable.

## Trial status

The study is currently ongoing. Recruitment began in August 2013 and will conclude in October 2014.

## Abbreviations

ACQ: Agoraphobic cognitions questionnaire
BDI-II: Beck Depression Inventory II
BSQ: Body sensations questionnaire
CBT: Cognitive behavioral therapy
CEA: Cost-effectiveness analysis
CES-D: Center for epidemiological studies depression scale
CONSORT: Consolidated standards of reporting trials
CUA: Cost-utility analysis
DRKS: German Clinical Trial Register
EMA: Ecological momentary assessment
EMGO: Institute for Health and Care Research
EMI: Ecologically momentary intervention
EQ-5D: EuroQol
HAM-A: Hamilton anxiety scale
ICER: Incremental cost-effectiveness ratio
INEP: Adverse effects of psychotherapy
MI: Mobility inventory
PAS: Panic and agoraphobia scale
PD: Panic disorder
QALYs: Quality-adjusted life years
RCI: Reliable change index
RCT: Randomized controlled trial
SCID-I: Structured clinical interview for DSM-IV Axis I Disorders
SF-12: Short form 12
SUS: System Usability Scale
TAM: Technology acceptance model
TiC-P: Trimbos and the Institute of Medical Technology Assessment Cost Questionnaire for Psychiatry
VU: Vrije Universiteit.
